# Preoperative Inflammatory Markers and Sonographic Free Peritoneal Fluid in Relation to Histopathologically Confirmed Appendiceal Perforation in Children: A Retrospective Cohort Study

**DOI:** 10.3390/children13091152

**Published:** 2026-08-27

**Authors:** Paul Tchouala Tchakoute, Răzvan Roșca, Alin Iuhas, Vlad-Ionuț Nechita, Cristian Phillip Marinău, Mihaela Ștefania Buzdugan, Ion Cosmin Puia

**Affiliations:** 1 Doctoral School of Biomedical Sciences, University of Oradea, 410073 Oradea, Romania; tchouala.paul@didactic.uoradea.ro (P.T.T.); cosmin.puia@umfcluj.ro (I.C.P.); 2 Department of Pediatric Surgery and Orthopedics, Bihor County Emergency Clinical Hospital, 410469 Oradea, Romania; 3 Department for the Prevention and Control of Healthcare-Associated Infections (CPIAAM), Clinical Hospital of Infectious Diseases and Pulmonology “Dr. Victor Babeș” Timișoara, Gheorghe Adam Street No. 13, 300310 Timișoara, Romania; 4 Department of Medical Disciplines, Faculty of Medicine and Pharmacy, University of Oradea, 410087 Oradea, Romania; 5 Department of Fundamental Sciences, Discipline of Medical Informatics, Research Methodology and Data Analysis, Faculty of Nursing and Health Sciences (FAMSS), “Iuliu Hațieganu” University of Medicine and Pharmacy, 400012 Cluj-Napoca, Romania; 6 Department of Surgery, “Octavian Fodor” Regional Institute of Gastroenterology and Hepatology, 400162 Cluj-Napoca, Romania; 7 Department of Surgery, “Iuliu Hațieganu” University of Medicine and Pharmacy, 400162 Cluj-Napoca, Romania

**Keywords:** appendicitis, intestinal perforation, child, leukocyte count, C-reactive protein, ultrasonography, logistic models, severity of illness index

## Abstract

Background/Objectives: Distinguishing perforated from non-perforated appendicitis preoperatively is difficult, and routine scores diagnose appendicitis rather than grade severity. We examined whether two inflammatory markers and one sonographic sign, combined in a model, discriminate between perforated and non-perforated appendicitis, and how this compares with a bedside score. Methods: Retrospective single-center cohort of 142 children aged 3–17 years undergoing initially attempted laparoscopic appendectomy (October 2022–December 2023). Log-transformed neutrophil-to-lymphocyte ratio (NLR) and C-reactive protein (CRP) were prespecified; sonographic free peritoneal fluid was selected from four signs using the outcome, and reported in-sample performance is an upper bound. Predictors were fitted by Firth penalized logistic regression with bootstrap validation. Results: Perforation occurred in 27 patients (19.0%). All three were independent predictors (odds ratios: CRP 2.583, NLR 3.045, free peritoneal fluid 3.638). The area under the curve was 0.900 (0.890 optimism-corrected). At the Youden-optimal threshold, sensitivity was 96.3% and negative predictive value 98.9%. The Pediatric Appendicitis Score reached 0.855 (difference not significant, *p* = 0.375); at matched sensitivity its specificity was 38.9% versus 74.8%. Conclusions: The model’s potential value lies in excluding perforation rather than establishing it, and applies after the decision to operate has been taken, informing antibiotic selection, operative urgency and family counselling rather than the indication for surgery. Overall discrimination did not differ significantly from the Pediatric Appendicitis Score, the advantage lying in the operating point rather than in the area under the curve. These findings are hypothesis-generating and require external validation.

## 1. Introduction

Acute appendicitis is the most common surgical emergency of childhood and the largest single indication for emergency abdominal surgery in patients under 18 years of age [1,2]. Global incidence in children was estimated at 109 per 100,000 per year in 2021 and increases with age through childhood [3]; in population-based US data, incidence peaks in those aged 10–19 years (23.3 per 10,000 per year), with a lifetime risk of approximately 6.7% in females and 8.6% in males [4]. Given this case volume, even modest improvements in preoperative severity stratification among children in whom appendicitis has been diagnosed would have broad relevance given the frequency of the condition.

Distinguishing perforated from non-perforated appendicitis before operation is clinically important because the two entities differ in management pathway, morbidity, and resource use. Complicated appendicitis carries a substantially higher postoperative complication rate than uncomplicated disease and is associated with longer hospital stay and increased rates of surgical-site infection and intra-abdominal abscess, particularly in perforated cases [5,6]. In adult cohorts, complicated appendicitis has also been linked to a greater likelihood of open or staged approaches and to the use of antibiotic-first management strategies, which are increasingly considered for selected patients [7]. Perforated appendicitis in children has been linked to a longer hospital stay: 4–13 versus 2–6 days in one pediatric series [8], and a median of 8 (interquartile range 7–11) versus 5 (4–7) days for complicated versus uncomplicated disease in a recent Romanian cohort [9]. In the present study, the outcome of interest is the narrower and histopathologically defined entity of appendiceal perforation rather than complicated appendicitis as a whole; evidence drawn from the broader complicated-appendicitis literature is flagged as such throughout. Preoperative knowledge of perforation does not alter the decision to operate: every child on this pathway undergoes appendectomy, and perforation is confirmed at operation and on histopathological examination. Its potential value lies elsewhere. Antibiotic management differs in kind rather than in degree between the two entities: uncomplicated appendicitis receives a single prophylactic dose before incision, whereas complicated disease is treated immediately with a therapeutic course continued for several days [5,6] (Section 2.5). Anticipating perforation may therefore inform whether a therapeutic course is started on admission rather than a single prophylactic dose, a decision taken before incision even though the definitive classification remains macroscopic and histopathological. Operative planning is affected in the same way, and families of children with perforation face a longer hospital stay [8,9] and a higher risk of postoperative complications [5,6]. A model of this kind is therefore intended for use after the decision to operate has been taken, and not for deciding whether an operation is indicated.

Reported perforation rates in pediatric series vary widely, from approximately 20% to 74%, reflecting differences in case mix, healthcare access, and diagnostic delay [6]. Age is a consistent modifier: rates are highest in the youngest children and fall progressively through adolescence [10], a gradient attributed to delayed or atypical presentation and diagnostic difficulty in younger children [11]. These observations underscore the value of population-specific rather than adult-derived estimates. A recent Western Romanian cohort reported a complicated-appendicitis rate of 32.6% at a median age of 11.1 years, within the 18–48% range described across Eastern Europe [9]. The inverse relationship between age and severity is established separately, in series reporting age-stratified perforation rates: 51% in children aged 5 years or younger, 32% at 6–9 years and 27% at 10 years or older in one large cohort [10], with delayed and atypical presentation in younger children advanced as the explanation [11].

Preoperative scores developed to diagnose appendicitis have performed less consistently when applied to the separate question of disease severity: the Alvarado score has been reported not to predict disease severity in children, although it retained some association with postoperative complications in the same study [12], and no scoring system for grading severity has achieved general acceptance [13]. Direct head-to-head comparisons of the same instrument on the two tasks in the same patients are, to our knowledge, not available. The Alvarado score and the Pediatric Appendicitis Score (PAS) were designed to identify appendicitis rather than to grade its severity [13,14], and the Appendicitis Inflammatory Response score, although useful for general risk stratification in suspected appendicitis [15], has shown limited accuracy for identifying high-risk pediatric patients who clearly require surgery [16]. Among laboratory markers, the white blood cell count alone discriminates poorly, whereas C-reactive protein (CRP) and the neutrophil-to-lymphocyte ratio (NLR) show a more consistent association with complicated disease [17,18], with proposed NLR cut-offs of 4.7 for appendicitis and 8.8 for complicated disease [19]. Imaging achieves high overall accuracy for diagnosing appendicitis in children, with ultrasound, computed tomography and magnetic resonance imaging all demonstrating excellent sensitivity and specificity and only modest differences between modalities [20,21,22]. A recognized limitation of ultrasound is that individual sonographic findings—such as free peritoneal fluid, loss of the submucosal echogenic layer and appendicolith—are highly specific for perforated appendicitis but variably sensitive, with reported specificities frequently above 85–90% and substantially lower sensitivities [23,24]. Combining findings into constellations further increases specificity, sometimes approaching 99–99.5%, at the cost of additional loss of sensitivity [24]. In real-world practice, negative or equivocal ultrasound examinations may not reliably exclude perforation, and false-negative studies have been reported [25].

Laboratory markers and sonographic findings have most often been evaluated as separate domains—either as blood-based indices such as the neutrophil-to-lymphocyte and platelet-to-lymphocyte ratios [17,18], or as ultrasound features [23,24]. A smaller number of pediatric studies have combined the two domains. Miyauchi et al. identified elevated C-reactive protein, together with ascites and appendicolith on imaging, among five independent predictors of perforation in a multivariable regression model [26]; Banerjee et al. reported that adding sonographic parameters to biochemical markers significantly improved specificity for complicated appendicitis [27]; and a machine-learning pipeline applied to multi-modal records distinguished perforated from non-perforated appendicitis in a cohort of 1980 children [28]. These reports differ in what they provide alongside the model. The two regression-based studies did not report internal validation of optimism, calibration, or a benchmark against a simple clinical score [26,27]. The machine-learning pipeline used split-sample validation and was compared against another severity-prediction tool, but did not report calibration, was not benchmarked against a bedside score, and relied on machine-learning infrastructure rather than a small set of routinely available variables [28]. None combined a deliberately minimal, transparent model with internal optimism correction, calibration, and a head-to-head comparison against an existing bedside score in the same patients.

The present study therefore does not claim novelty for the combination of modalities as such. It examines how a deliberately minimal three-variable combination—preoperative log-transformed NLR, log-transformed C-reactive protein, and sonographic free peritoneal fluid—relates to histopathologically confirmed appendiceal perforation in children aged 3–17 years undergoing an initially attempted laparoscopic appendectomy at a single center, when that combination is internally validated, calibrated, and benchmarked in the same patients against the Pediatric Appendicitis Score [29,30]. The analysis is retrospective and hypothesis-generating, and is not intended to deliver a validated preoperative tool.

## 2. Materials and Methods

### 2.1. Study Design and Setting

This retrospective observational cohort study was carried out in the Department of Pediatric Surgery and Orthopedics, Bihor County Emergency Clinical Hospital, Oradea, Romania. Patients were identified across 15 consecutive months, between October 2022 and December 2023. Reporting follows the STROBE (Strengthening the Reporting of Observational Studies in Epidemiology) statement for cohort studies; the completed checklist is provided as File S1.

The same cohort of 142 patients has been analyzed in a companion manuscript, now published in Surgeries (Section 4.6), which differs from the present study in four respects. Its objectives concern different primary outcomes, namely conversion from a laparoscopic to an open approach and peritonitis assessed intraoperatively, rather than histopathologically confirmed perforation. Its predictor set overlaps only partly with the one used here. Its analytical approach is receiver operating characteristic analysis of standardized composite scores, rather than penalized regression with internal validation and calibration. Its clinical conclusions concern intraoperative findings and operative course, not preoperative risk stratification for perforation. The two reports share only the descriptive characterization of the cohort; no inferential result is duplicated.

### 2.2. Participants

Of the 185 children assessed for suspected acute appendicitis over the study period, 43 fell outside the scope of the present analysis: 7 were managed without operation and 36 proceeded directly to an open procedure. The analyzed sample therefore comprised the remaining 142 children, in each of whom laparoscopy was the operative approach chosen at the outset.

Inclusion criteria were: age between 3 and 18 years; appendicitis documented both at operation and on histopathological examination; laparoscopy as the operative approach chosen at the outset; and complete clinical and operative records. The ages actually observed spanned 3 to 17 years. Exclusion criteria were: management without surgery; an open procedure planned from the start; and removal of the appendix incidental to a different operation. The cohort was defined a priori around the initially attempted laparoscopic pathway. Children treated conservatively were excluded because the outcome required histopathological examination of a resected specimen; children undergoing primary open appendectomy (n = 36) fell outside this predefined pathway and were not analyzed. The measurements themselves did not depend on the operative approach, since all predictors were obtained preoperatively and the outcome on the resected specimen; whether the restriction altered the case mix of the analyzed sample is addressed in Section 4.6. Patients converted to an open approach during surgery (n = 7) were retained in the cohort.

### 2.3. Outcome Definition

The primary outcome was appendiceal perforation, determined by histopathological examination of the resected specimen. Specimens were graded on an ordinal scale of increasing severity as catarrhal/congestive, phlegmonous, gangrenous, or perforated. The outcome was defined as a binary variable, with the perforated category coded as the event and the remaining three categories as non-events. Histopathological assessment was performed at the Department of Pathology, Bihor County Emergency Clinical Hospital, 410469, Oradea, Romania, as part of routine clinical care, independently of the present analysis.

Histopathological grading followed the routine diagnostic criteria applied in the department: perforation was defined as full-thickness disruption of the appendiceal wall on microscopic examination. Reports were issued by the attending pathologist as part of clinical care; no independent re-review or inter-observer agreement assessment was performed for the present analysis, and the number of sections examined per specimen was not standardized. The implications of this for outcome misclassification are addressed in Section 4.6.

### 2.4. Predictor Variables

Three predictors were entered into the multivariable model: two systemic inflammatory markers and one sonographic sign. The neutrophil-to-lymphocyte ratio and C-reactive protein were specified in advance on the basis of biological rationale and previous literature. The sonographic predictor was selected from among the four recorded signs on the basis of the exploratory analysis reported in Section 3.2, which was performed before the multivariable model was fitted. No automated or stepwise selection procedure was applied at any stage, and the predictor set was not modified after the model had been fitted.

Although no automated selection algorithm was used, the sonographic predictor was chosen on the basis of its association with the outcome in the same dataset. This data-driven step introduces a source of optimism that resampling the final model alone would not capture. The internal validation described in Section 2.6.4 therefore repeated the selection step within each bootstrap replicate, so that the optimism estimate reflects the procedure that generated the model rather than the model taken as given. Even after this correction, the reported performance should be regarded as an upper bound, because the model was both developed and evaluated in the same cohort and the operating threshold was optimized in-sample—sources of optimism that the bootstrap, which addresses only the selection of the sonographic sign, does not remove.

Neutrophil-to-lymphocyte ratio. Derived from the complete blood count obtained at admission, as the absolute neutrophil count divided by the absolute lymphocyte count.

C-reactive protein. Measured at admission, expressed in mg/L.

Free peritoneal fluid on ultrasound. A binary variable extracted from the preoperative abdominal ultrasound report, coded as present or absent.

Both inflammatory markers were markedly right-skewed (Shapiro–Wilk W = 0.921 for NLR and W = 0.748 for CRP, both *p* < 0.001) and were entered into the regression model after natural logarithmic transformation. CRP was transformed as log(CRP + 1), the constant being added to preserve values close to zero (observed minimum 0.03 mg/L). NLR was log-transformed without an added constant (observed minimum 0.58).

The Pediatric Appendicitis Score was recorded but was not entered into the multivariable model, for two reasons. First, the score is a composite that already incorporates leukocytosis and neutrophilia and therefore overlaps conceptually with the inflammatory markers under study. Second, the number of outcome events restricted the model to three degrees of freedom (Section 2.6.2). The score was retained as a comparator in the diagnostic performance analysis, where it does not consume model degrees of freedom.

#### Ultrasound Assessment

All patients underwent preoperative abdominal ultrasound as part of routine diagnostic evaluation. Examinations were performed by the radiologists on duty in the hospital’s radiology department, using a Samsung RS80 EVO ultrasound system (Samsung Medison, Seoul, Republic of Korea) equipped with convex (CA1-7A) and linear (L3-12A) transducers. No study-specific protocol or structured reporting template was applied; sonographic findings were extracted from the clinical reports issued at the time of the examination.

Four sonographic signs were recorded as binary variables: appendix visualization, appendiceal wall thickening, free peritoneal fluid, and appendicolith.

### 2.5. Additional Variables

Demographic, anthropometric, laboratory, and perioperative variables were abstracted from the hospital information system, from the operative reports, and from the discharge documentation. Body mass index was derived from weight and height as kg/m^2^ and treated as a continuous variable. Every anesthetic chart recorded American Society of Anesthesiologists (ASA) physical status 1, and no chronic comorbidity appeared in any record. Given that the cohort included 27 children with histopathologically confirmed perforation, of whom 24 had generalized peritonitis recorded at operation, a uniform ASA 1 classification is more plausibly a feature of how this field was completed in routine practice than an accurate reflection of preoperative physical status. The variable was constant and therefore carried no information for any analysis; it was set aside, and is noted here as an indication of the granularity of the source documentation.

Perioperative management. All operations were performed under general anesthesia with orotracheal intubation; a nasogastric tube was placed when the stomach was full. Perioperative antibiotic use was standardized within the department and distinguished uncomplicated from complicated appendicitis. Where appendicitis was judged uncomplicated, a single intravenous dose of ceftriaxone 50 mg/kg with metronidazole 15 mg/kg was given 60 min before incision, and no postoperative antibiotics were administered. Where appendicitis was complicated, therapeutic treatment was started immediately rather than as single-dose prophylaxis, using ceftriaxone 50 mg/kg/day with metronidazole 30 mg/kg/day, or meropenem 60 mg/kg/day, for three to five days, followed where required by oral amoxicillin-clavulanate 45 mg/kg/day. Laparoscopy was performed through three ports in every patient; the appendiceal stump was closed with two Roeder loops and the specimen retrieved through a trocar. Drainage was placed at the discretion of the operating surgeon. The 36 children who underwent primary open appendectomy during the same period did so because of anesthetic contraindications or technical considerations.

### 2.6. Statistical Analysis

Analyses were performed in R version 4.6.1 (R Foundation for Statistical Computing, Vienna, Austria), using the packages logistf for penalized regression, pROC for receiver operating characteristic analysis, rms for the restricted cubic spline assessment, calibration and internal validation, and ResourceSelection for goodness-of-fit testing. A two-sided *p*-value below 0.05 was considered statistically significant.

No adjustment for multiple comparisons was applied. The baseline comparisons in Table 1 are descriptive. The four sonographic comparisons, by contrast, determined which sign entered the multivariable model; the resulting selection effect is addressed by reporting the effective events-per-variable ratio (Section 2.6.2) and by repeating the selection inside the bootstrap validation (Section 2.6.4). All reported *p*-values should be read as nominal.

#### 2.6.1. Descriptive Statistics

Normality was assessed using the Shapiro-Wilk test. All continuous variables except the leukocyte count deviated significantly from normality, and all were therefore summarized as median [interquartile range] and compared using the Mann-Whitney U test. Categorical variables are expressed as counts and percentages; sex was compared using the chi-squared test with continuity correction, and the four sonographic signs using Fisher’s exact test, applied uniformly across this group because one sign had expected cell counts below five. Standardized mean differences are additionally reported.

#### 2.6.2. Sample Size and Events per Variable

No a priori sample size calculation was performed; the study included all consecutive eligible patients within the defined period. With 27 outcome events, the model was constrained a priori to three degrees of freedom, corresponding to a nominal events-per-variable (EPV) ratio of 9.0.

This nominal ratio understates the burden placed on the data. Two predictors were specified in advance, but the third was chosen from among four recorded sonographic signs on the basis of their association with the outcome in this same sample. Counting the six parameters exposed to the data in total gives an effective events-per-variable ratio of 4.5, a range in which overfitting and small-sample bias are a recognized concern [31]. The internal validation results should be read against the effective rather than the nominal figure.

Applying the sample size approach of Riley et al. [31] post hoc to the fitted model (three parameters, outcome proportion 0.190, Cox-Snell R2 of 0.312) gives minimum sample sizes of 71 and 79 for the criteria limiting expected shrinkage and the difference between apparent and optimism-adjusted R2, both satisfied by the 142 patients available, and of 237 for the criterion concerning precise estimation of the overall outcome risk, which is not met.

#### 2.6.3. Regression Model

The primary multivariable model was fitted using Firth’s penalized maximum likelihood logistic regression, which reduces small-sample bias and yields finite estimates under separation. Confidence intervals and *p*-values were derived from profile penalized likelihood rather than Wald statistics, which are unreliable at small event counts. A conventional maximum likelihood model was fitted to the same data as a sensitivity analysis (Table S1).

Three assumptions were examined before the model was interpreted: separation, by inspection of unpenalized coefficients and standard errors in the initially specified model; multicollinearity, by variance inflation factors and Spearman rank correlation between the continuous predictors; and linearity of the logit for the neutrophil-to-lymphocyte ratio, by restricted cubic splines with three knots at the 10th, 50th and 90th percentiles, the non-linear terms being tested by likelihood ratio. The results are reported in Section 3.3 and supported modelling the ratio on the logarithmic scale; this decision rested on the distributional properties and the linearity assumption, not on model performance.

Odds ratios for log-transformed predictors are additionally reported per doubling of the original variable, calculated as exp(β × ln 2), to facilitate clinical interpretation.

#### 2.6.4. Model Performance and Validation

Discrimination was assessed by the area under the receiver operating characteristic curve (AUC) with 95% confidence intervals. Cut-off values were determined by maximizing Youden’s index, with the corresponding sensitivity, specificity and predictive values; because they were derived from the development data, they represent optimal values within this cohort. AUCs were compared using DeLong’s test for correlated curves. Because the intended use of such a model is to exclude rather than to confirm perforation, specificity and predictive values were additionally compared at a matched sensitivity in the high-sensitivity region; this operating-point comparison was prompted by the clinical question rather than pre-specified, and is reported as exploratory.

Calibration was evaluated using the Hosmer–Lemeshow goodness-of-fit test with five and ten groups, and by graphical inspection of the calibration curve with bootstrap bias correction; the mean absolute difference between predicted and observed probabilities is reported.

Internal validation was performed by bootstrap resampling with 1000 replications, yielding optimism-corrected estimates of discrimination, explained variation and the calibration slope. Because the resampling routine does not support the penalized-likelihood implementation used for the primary model, it was applied to a conventional maximum likelihood model with the same predictors; the two fits produced closely comparable coefficients (Table 2 and Table S1) and identical apparent discrimination to three decimal places, so the optimism estimates are reported as applying to the penalized model to a close approximation.

Because the sonographic predictor was selected using the outcome (Section 2.4), resampling the final model alone would not capture the optimism generated by that selection. The bootstrap was therefore run in two versions: in the first the three predictors were held fixed; in the second the full procedure was repeated within each replicate, the four sonographic signs being re-screened and the most strongly associated carried forward. The second version is reported as the primary internal validation result.

No external validation was undertaken, and the model is not presented as a validated prediction tool.

#### 2.6.5. Exploratory Analyses

The four individual sonographic signs were examined separately in relation to the outcome using Fisher’s exact test; odds ratios and 95% confidence intervals are those produced by the conditional maximum likelihood estimator implemented in that test. These analyses preceded the multivariable model and informed the choice of the sonographic predictor (Section 2.4). They are reported in full, including null findings.

#### 2.6.6. Missing Data

The outcome and all three model predictors were complete for all 142 patients; no imputation was required and the multivariable analysis was performed on the full cohort. The Pediatric Appendicitis Score was missing for two patients (1.4%) and was analyzed on available cases (n = 140).

### 2.7. Ethical Considerations

The study was conducted in accordance with the Declaration of Helsinki and approved by the Ethics Committee of the Bihor County Emergency Clinical Hospital, Oradea, Romania (approval no. 24548, 28 July 2026). Patient consent was waived owing to the retrospective design of the study. All data were anonymized prior to analysis.

## 3. Results

### 3.1. Cohort Characteristics

**Figure 1:**
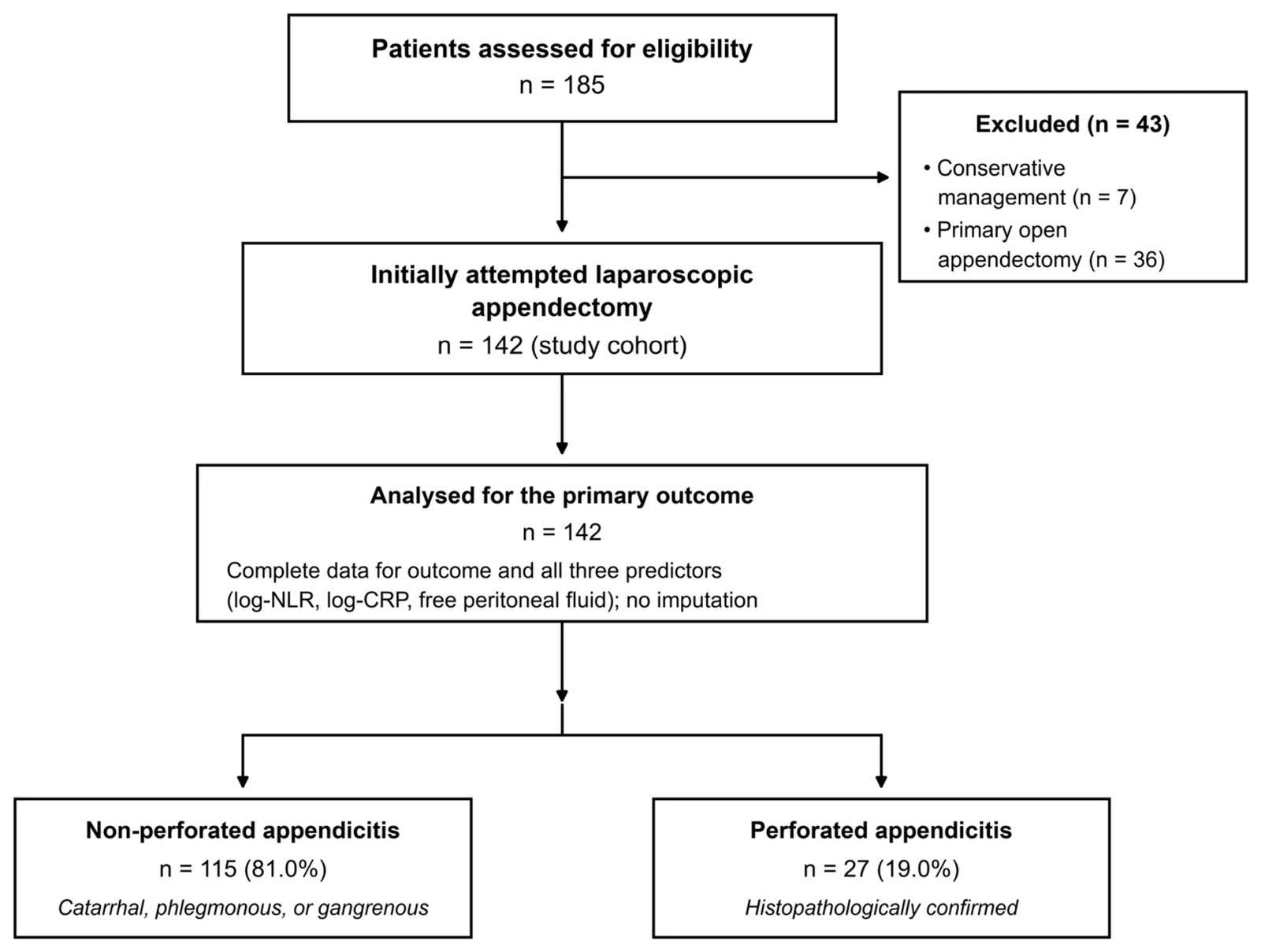
Study flowchart. Of 185 pediatric patients assessed for eligibility between October 2022 and December 2023, 43 were excluded (conservative management, n = 7; primary open appendectomy, n = 36) and 142 underwent an initially attempted laparoscopic appendectomy, constituting the study cohort. Seven patients converted to an open approach during surgery were retained, since neither the predictors nor the outcome depended on the operative approach. The outcome was determined by histopathological examination of the resected specimen. Data were complete for the outcome and all three predictors and no imputation was required; the Pediatric Appendicitis Score was missing for two patients and was analyzed on available cases. The events-per-variable ratio is given both as the nominal value, counting the three predictors entered, and as the effective value, counting the four sonographic signs screened to select one of them (Section 2.6.2). CRP, C-reactive protein; NLR, neutrophil-to-lymphocyte ratio.

Of 185 pediatric patients assessed for suspected acute appendicitis during the study period, 142 underwent an initially attempted laparoscopic appendectomy and constituted the study cohort (Figure 1). On histopathological examination, 39 specimens (27.5%) were graded as catarrhal or congestive, 55 (38.7%) as phlegmonous, 21 (14.8%) as gangrenous and 27 (19.0%) as perforated. Appendiceal perforation, the primary outcome, was therefore confirmed in 27 patients (19.0%); the 21 gangrenous specimens were classified as non-events. Taking the broader category of complicated appendicitis—gangrenous or perforated disease, or peritonitis recorded at operation—51 patients (35.9%) were affected, and peritonitis of any grade was recorded in 40 (28.2%).

Seven patients (4.9%) were converted from a laparoscopic to an open approach during surgery and were retained in the cohort. Perforation was confirmed in 6 of these 7 (85.7%), compared with 21 of the 135 not converted (15.6%; Fisher’s exact test, *p* < 0.001), so that 6 of the 27 perforations (22.2%) occurred in patients requiring conversion. Refitting the multivariable model after excluding these seven patients left the estimates materially unchanged (n = 135, 21 events; log-NLR odds ratio 2.784, log-CRP 2.476, free peritoneal fluid 3.847; area under the curve 0.895).

Baseline characteristics of the cohort, stratified by perforation status, are presented in Table 1. Age (median 11.00 years, interquartile range 9.00–14.00) did not differ between patients with and without perforation (10.00 [7.50–14.00] versus 11.00 [9.00–14.00]; *p* = 0.605), nor did sex distribution (female sex 37.0% versus 39.1%; *p* = 1.000) or body mass index (21.13 [15.99–22.84] versus 19.39 [16.53–22.21] kg/m^2^; *p* = 0.662).

All four inflammatory parameters differed significantly between groups. The largest separation was observed for C-reactive protein, whose median was approximately nine to ten times higher in patients with perforation (89.20 [46.85–130.00] versus 9.40 [1.70–34.50] mg/L; *p* < 0.001; standardized mean difference 1.241). The neutrophil-to-lymphocyte ratio was likewise higher (9.91 [7.33–13.50] versus 5.25 [2.80–9.53]; *p* < 0.001). Differences in the neutrophil count (12.57 [11.49–15.44] versus 10.80 [5.80–13.27] × 10^9^/L; *p* = 0.004) and the leukocyte count (14.88 [13.77–17.64] versus 13.40 [9.04–16.74] × 10^9^/L; *p* = 0.023) were smaller in magnitude. The Pediatric Appendicitis Score was higher in patients with perforation (9.00 [8.00–10.00] versus 7.00 [6.00–8.00]; *p* < 0.001), with the largest standardized mean difference observed in the cohort (1.576).

### 3.2. Sonographic Findings

Among the four sonographic signs recorded, only free peritoneal fluid was associated with perforation. It was present in 48.1% of patients with perforation compared with 16.5% of those without (odds ratio 4.63, 95% confidence interval (CI) 1.71–12.63; Fisher’s exact test, *p* = 0.001).

The remaining three signs showed no association with the outcome. Appendix visualization occurred with almost identical frequency in both groups (66.7% versus 66.1%; odds ratio 1.03, 95% CI 0.39–2.85; *p* = 1.000), as did appendiceal wall thickening (44.4% versus 47.0%; odds ratio 0.90, 95% CI 0.35–2.28; *p* = 0.834). An appendicolith was identified in only four patients in the entire cohort (2.8%), and the corresponding estimate was correspondingly imprecise (odds ratio 1.43, 95% CI 0.03–18.67; *p* = 0.574).

Because only one of the four signs was associated with the outcome, the signs were not combined into an aggregate score. In accordance with the procedure described in Section 2.4, free peritoneal fluid was the sonographic sign carried forward into the multivariable model; the other three signs were not considered further.

### 3.3. Multivariable Model

The multivariable Firth penalized logistic regression model included three predictors and was fitted on the complete cohort of 142 patients with 27 events (nominal events per variable 9.0; effective 4.5, Section 2.6.2). Diagnostic checks performed before interpretation showed no evidence of separation (maximum standard error 1.12 in the initially specified model), negligible collinearity in the final model (all variance inflation factors below 1.03; Spearman ρ = 0.21 between the two log-transformed markers), and no violation of the linearity assumption after logarithmic transformation of the neutrophil-to-lymphocyte ratio (restricted cubic spline non-linearity test *p* = 0.267, compared with *p* = 0.044 on the untransformed scale).

All three predictors were independently associated with perforation (Table 2). The log-transformed C-reactive protein carried the strongest association (odds ratio 2.583, 95% CI 1.733–4.213; *p* < 0.001), corresponding to an odds ratio of 1.931 (95% CI 1.464–2.710) per doubling of the original value. The log-transformed neutrophil-to-lymphocyte ratio was also associated with the outcome (odds ratio 3.045, 95% CI 1.381–7.547; *p* = 0.005), equivalent to an odds ratio of 2.164 (95% CI 1.251–4.059) per doubling. The presence of free peritoneal fluid was associated with an odds ratio of 3.638 (95% CI 1.215–11.375; *p* = 0.021), although the confidence interval was wide, reflecting the number of patients in whom this sign was present.

The model was significant overall (likelihood ratio χ^2^ = 50.45, df = 3, *p* < 0.001), with a Nagelkerke R^2^ of 0.502 and a Brier score of 0.093.

A conventional maximum likelihood model fitted to the same data yielded estimates consistent in direction and magnitude (log-NLR odds ratio 3.307, log-CRP 2.776, free peritoneal fluid 3.872; all *p* ≤ 0.021; Table S1). The slight attenuation of the penalized estimates towards the null is the expected consequence of penalization and indicates that estimation was stable in this dataset.

### 3.4. Diagnostic Performance

**Figure 2:**
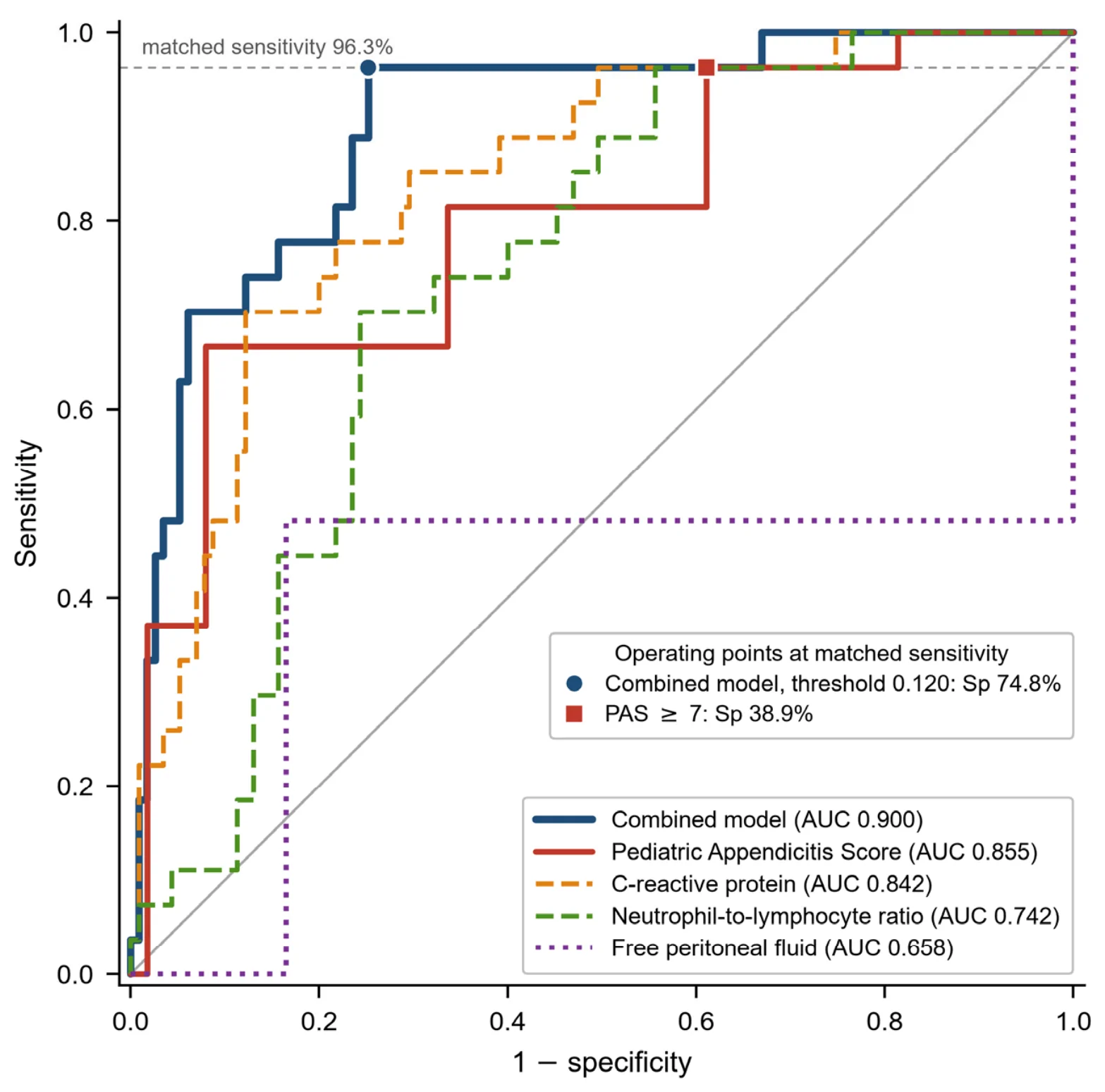
Receiver operating characteristic curves for the prediction of appendiceal perforation. The combined model (log-NLR, log-CRP, and free peritoneal fluid) achieved an AUC of 0.900 (95% CI 0.839–0.963), higher than each of its three individual components, as is inevitable for a model fitted to those components in the same sample. The difference versus the Pediatric Appendicitis Score (AUC 0.855), an external comparator, was not statistically significant (*p* = 0.375; Table 4). The horizontal dashed line marks the matched sensitivity of 96.3%; at this sensitivity the combined model (filled circle; predicted-probability threshold 0.120) reached a specificity of 74.8% and the Pediatric Appendicitis Score (filled square; PAS ≥ 7) a specificity of 38.9%. These matched-sensitivity operating points were computed post hoc within the development sample and are exploratory (Section 3.4; note to Table 3). The diagonal reference line denotes chance-level discrimination. AUC, area under the curve; CRP, C-reactive protein; NLR, neutrophil-to-lymphocyte ratio; PAS, Pediatric Appendicitis Score; Sp, specificity.

The discriminative performance of the individual parameters and of the combined model is presented in Table 3 and Figure 2.

The combined model achieved an area under the curve of 0.900 (95% CI 0.839–0.963). At the cut-off maximizing Youden’s index (predicted probability 0.120), it yielded a sensitivity of 96.3% and a negative predictive value of 98.9%, with a specificity of 74.8% and a positive predictive value of 47.3%. At this threshold, one of the 27 perforated cases was classified below the cut-off. Because this sensitivity corresponds to 26 of 27 events, the operating point is inherently unstable: misclassification of a single additional perforation would reduce sensitivity to 92.6% and lower the negative predictive value accordingly. These operating-point estimates were not accompanied by confidence intervals, and their apparent precision should be interpreted with corresponding caution.

Among individual parameters, the Pediatric Appendicitis Score showed the highest discrimination (area under the curve 0.855, 95% CI 0.774–0.936; cut-off ≥ 8.5; sensitivity 66.7%, specificity 92.0%), followed by C-reactive protein (0.842, 95% CI 0.763–0.920; cut-off ≥ 65.2 mg/L; sensitivity 70.4%, specificity 87.8%) and the neutrophil-to-lymphocyte ratio (0.742, 95% CI 0.652–0.832; cut-off ≥ 9.66; sensitivity 70.4%, specificity 75.7%). Free peritoneal fluid alone showed the lowest discrimination (0.658, 95% CI 0.556–0.760), with a sensitivity of 48.1% and a specificity of 83.5%.

Pairwise differences in the area under the curve are shown in Table 4. Because the combined model was fitted to these same three predictors, it discriminates at least as well as each of them in-sample by construction, and the corresponding comparisons are reported for completeness rather than as tests of an independent hypothesis. The magnitude of the difference is nonetheless informative and varied markedly: 0.242 (95% CI 0.150–0.335) versus free peritoneal fluid and 0.158 (95% CI 0.067–0.250) versus the neutrophil-to-lymphocyte ratio, but only 0.059 (95% CI 0.009–0.109) versus C-reactive protein. The comparison that does test an independent question—against the Pediatric Appendicitis Score—is not significant (Section 4.4).

In the 140 patients with an available Pediatric Appendicitis Score, the combined model yielded an area under the curve of 0.899 compared with 0.855 for the score. This difference of 0.044 was not statistically significant (95% CI −0.053 to 0.141; *p* = 0.375). The confidence interval spans zero and is compatible with a moderate advantage for either measure.

The two measures nonetheless occupied different operating points, and comparing them at a common sensitivity is more informative than comparing areas. At its Youden-optimal threshold (≥8.5), the score achieved a sensitivity of 66.7% and missed 9 of the 27 perforations, whereas the model at its own optimal threshold achieved 96.3% and missed 1. Lowering the score threshold to ≥7 matched the model’s sensitivity of 96.3%, with the same single missed perforation, but at a specificity of 38.9% compared with 74.8% for the model. Expressed in absolute terms, at a sensitivity of 96.3% the model correctly classified 86 of the 115 patients without perforation as low risk, whereas the score correctly classified 44 of 113. Any threshold of the score reaching 100% sensitivity (≤6) did so at a specificity of 18.6% or lower.

### 3.5. Calibration and Internal Validation

**Figure 3:**
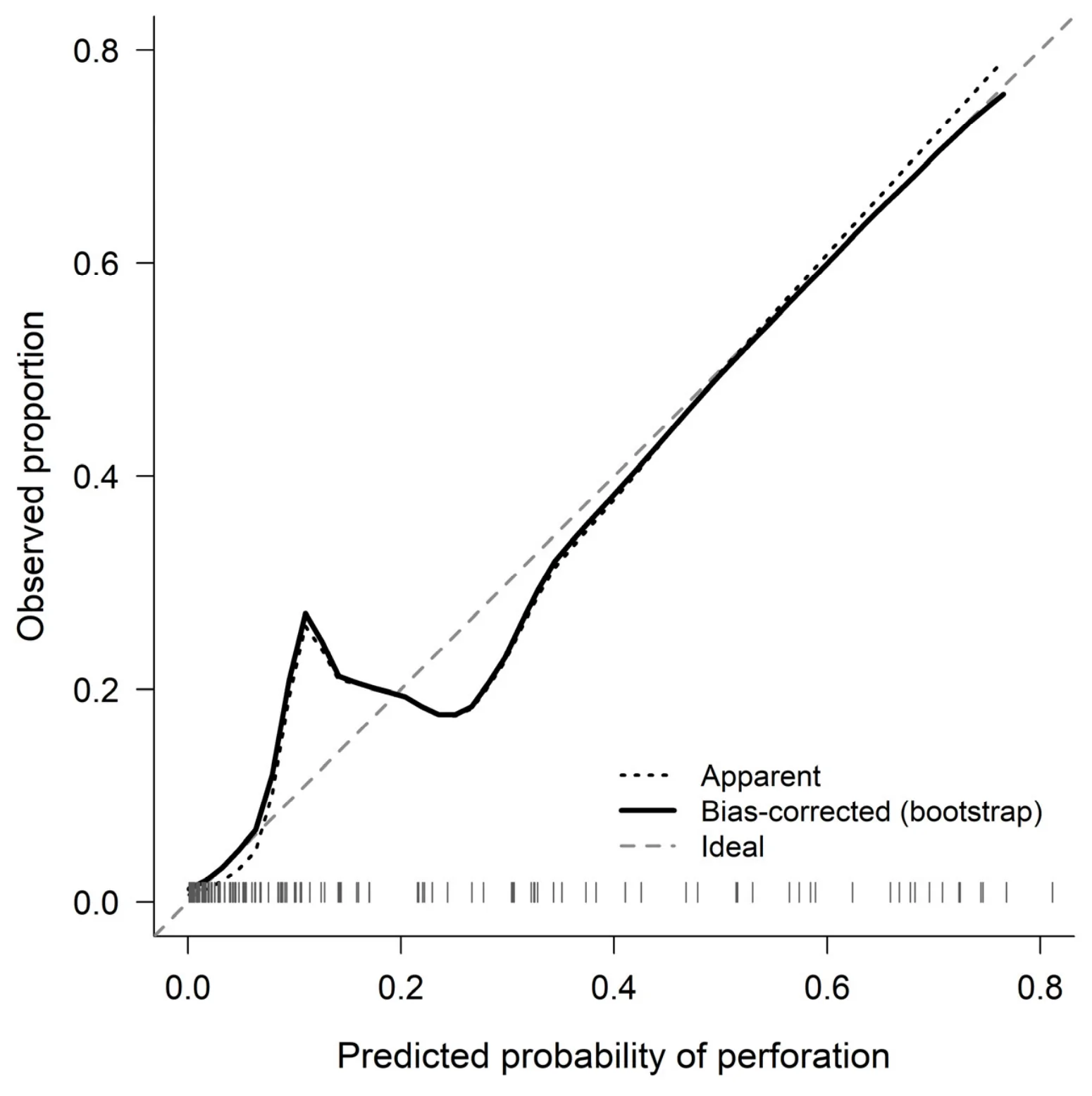
Calibration curve of the corresponding unpenalized model (Section 2.6.4) with bootstrap bias correction (1000 replications). The bias-corrected curve nearly overlapped the apparent curve, consistent with the small optimism observed on internal validation (0.010). The dashed diagonal represents perfect calibration. Mean absolute error 0.026. Tick marks along the horizontal axis indicate the distribution of predicted probabilities.

Calibration of the combined model was adequate. The Hosmer–Lemeshow test, applied to the predicted probabilities of the penalized model, showed no evidence of lack of fit with either ten groups (*p* = 0.212) or five groups (*p* = 0.425). The calibration curve with bootstrap bias correction is shown in Figure 3; the mean absolute difference between predicted and observed probabilities, computed on the corresponding unpenalized model used for the bootstrap (Section 2.6.4), was 0.026. The bias-corrected and apparent curves were nearly superimposed across the range of predicted values. The largest departures from the ideal line occurred at low predicted probabilities: around the Youden-optimal threshold (predicted probability approximately 0.12) the curve lay above the ideal line, the model underestimating the observed proportion of perforation, whereas between predicted probabilities of approximately 0.20 and 0.30 it modestly overestimated it. Above a predicted probability of 0.30 the curve tracked the ideal line closely.

Internal validation by bootstrap resampling with 1000 replications, performed on the corresponding unpenalized model as described in Section 2.6.4, yielded an optimism-corrected area under the curve of 0.890, compared with an apparent value of 0.900. The optimism-corrected calibration slope was 0.924.

The two versions of the bootstrap gave closely similar results. Holding the three predictors fixed, the estimated optimism was 0.008. Repeating the selection of the sonographic sign within each replicate, which is the procedure that generated the model and the value reported above, gave an optimism of 0.010. The cost of the selection step was therefore approximately 0.002 on the AUC scale. This small difference has a straightforward explanation: free peritoneal fluid was the most strongly associated of the four signs in 94.6% of bootstrap replicates, so that the selection behaved almost deterministically and contributed little additional instability. The small optimism estimate indicates limited overfitting in this dataset; however, internal validation cannot substitute for evaluation in an independent cohort, and the model is not presented as externally validated.

### 3.6. Representative Imaging and Operative Findings

**Figure 4:**
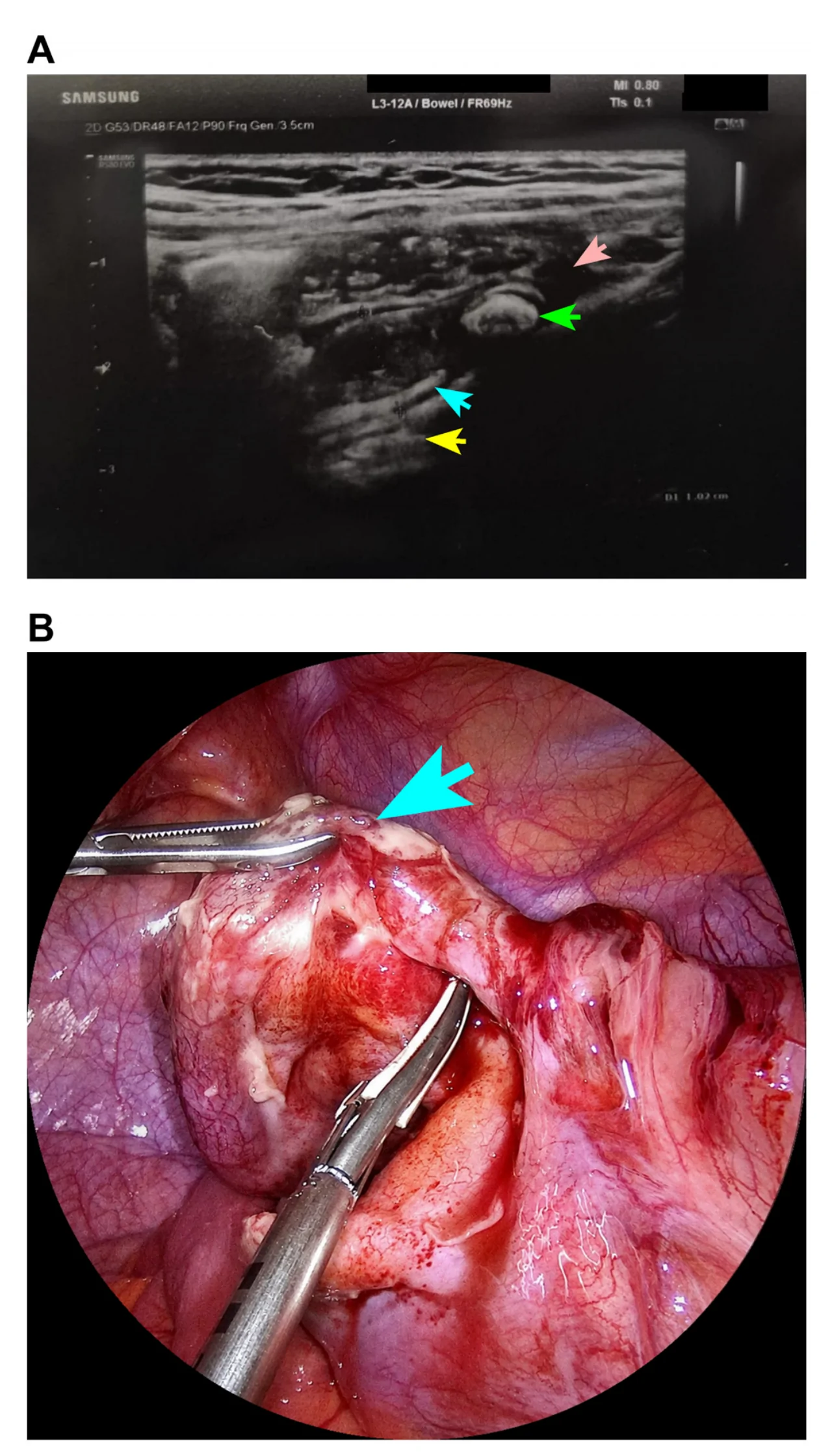
Representative preoperative sonographic and intraoperative laparoscopic findings in perforated appendicitis. (**A**) Sonographic image of the right lower quadrant, obtained with the linear L3-12A transducer (Section Ultrasound Assessment), showing an enlarged appendix with a maximum diameter of 1.02 cm (cyan arrow), a luminal appendicolith (green arrow), adjacent free peritoneal fluid (pink arrow), and increased echogenicity of the periappendiceal fat (yellow arrow). (**B**) Intraoperative laparoscopic view of the perforated appendix (cyan arrow), with gangrenous wall changes and associated peritonitis. The panels are from different patients. Patient identifiers have been removed.

Figure 4 illustrates the sonographic and intraoperative appearance of perforated appendicitis in two representative patients from the cohort. These images are illustrative and were not part of the analysis.

## 4. Discussion

### 4.1. Principal Findings

In this retrospective cohort of 142 children undergoing an initially attempted laparoscopic appendectomy, each of the three prespecified predictors was independently associated with histopathologically confirmed perforation, and their combination discriminated well after correction for optimism. This lies toward the higher end of values reported for preoperative discrimination of perforation in pediatric appendicitis, although no systematic review was undertaken and the comparison is illustrative rather than exhaustive. A three-variable clinical scoring system based on duration of symptoms, fever and absolute neutrophil count achieved an AUC of 0.89 for perforation [32], and a rule-in risk-stratification model reported 0.78 for the same outcome [33]. The most directly comparable report is a machine-learning pipeline developed in 1980 children with a perforation rate of 30.4%, which reached a negative predictive value of 82.8% and a positive predictive value of 56.4% [28]; the present model achieved a higher apparent negative predictive value and a lower positive predictive value, but because both depend on prevalence (19.0% here), that difference is in part a mechanical consequence of the lower event rate rather than of superior discrimination. A further machine-learning study reported AUC values up to 0.91, but for postoperative complications, with perforation entering as a predictor rather than as the outcome [34].

The performance of the model was asymmetric: it is more useful for excluding perforation than for confirming it. Both predictive values are properties of this cohort at a perforation prevalence of 19.0% and at a threshold chosen within these same data, and both would change in a population with a different prevalence.

The observed perforation rate of 19.0% (27/142) lies just below the lower bound of the range commonly reported in pediatric appendicitis series (approximately 20–74%, with rates most often quoted around 30%) [6]. It is not explained by the age structure of the cohort. Series with a comparable age distribution report substantially higher rates: 27% in children aged 10 years or older [10], 30–40% in children over 8 years [6], 31.5% in a cohort with a median age of 11 years [8], and 30.4% at a mean age of 10.7 years [28].

The most plausible explanation is definitional, and the present data allow it to be quantified rather than merely asserted; selective exclusion of children undergoing primary open appendectomy, considered below, may also contribute. The published range is drawn from series in which perforation was identified macroscopically [6], whereas the present outcome required histopathological confirmation of full-thickness wall disruption. Under the broader definition used in most of those series, 51 of the 142 patients (35.9%) had complicated appendicitis and peritonitis of any grade was present in 40 (28.2%)—figures that sit within the published range and match the 32.6% complicated-appendicitis rate reported at a comparable median age in the Western Romanian cohort cited above [9]. The cohort is therefore not unusual in severity; the outcome definition is narrower. Twenty-one children with gangrenous appendicitis, a category that behaves clinically much like perforation, were classified as non-events throughout this analysis.

A second consideration operates in the same direction and is taken up in the Limitations: 36 children underwent primary open appendectomy during the same period and were not analyzed, and if that approach was chosen more often in children with a more advanced presentation, the analyzed sample is depleted of severe cases. The two explanations are not alternatives; both would act simultaneously and in the same direction.

### 4.2. What the Three Predictors Contribute

Because the combined model was fitted to these three predictors, it discriminates at least as well as each of them in-sample by construction, and the pairwise significance tests against its own components test no independent hypothesis. What is informative is the magnitude of the gain, which differed markedly across comparisons (Table 4).

Both inflammatory markers have been examined previously in relation to complicated appendicitis in children. A systematic review and meta-analysis reported that the neutrophil-to-lymphocyte ratio can help distinguish complicated from uncomplicated appendicitis, with proposed cut-off values of 4.7 and 8.8 [19]; complicated appendicitis is a broader category than the endpoint used here and the cut-offs are not directly transferable, although the threshold identified in the present cohort is close to the 8.8 proposed for the broader entity. In pediatric cohorts, elevated NLR has also been associated with perforation specifically, with more modest and variable reported performance [18]. C-reactive protein has been associated with perforated appendicitis in pediatric cohorts, appearing among five independent predictors in one retrospective study [26], and CRP-based ratios such as the CRP-to-prealbumin ratio have been proposed to aid discrimination [35].

In the present cohort the two markers were only weakly correlated and collinearity was negligible, so neither estimate was destabilized by the presence of the other. Low collinearity does not by itself establish that the two carry non-redundant information about the outcome; the relevant evidence is the increment in discrimination reported in Table 4.

The sonographic sign and the serum markers reflect complementary aspects of the disease process: free peritoneal fluid is a local morphological finding, whereas serum inflammatory markers reflect the systemic inflammatory response [27]. Free peritoneal fluid is not specific to perforation and may contribute to the sonographic diagnosis mainly when interpreted in combination with other findings [24]; in the present cohort, it was present in 19 of 115 non-perforated cases. Its contribution to the model therefore indicates an association with perforation rather than a deterministic relationship [24].

Neither marker is specific to perforation: both reflect the systemic inflammatory response rather than appendiceal wall disruption, which is expected, since perforation is accompanied by rather than independent of a rising inflammatory burden. The relevant question is therefore whether their combination with a local sonographic sign improves preoperative stratification over simpler alternatives, which Section 4.4 addresses directly.

### 4.3. Individual Sonographic Signs

As reported in Section 3.2, free peritoneal fluid was the only one of the four signs associated with perforation. This is consistent with the recognized limitations of ultrasound in distinguishing perforated from non-perforated appendicitis, where reported sensitivity is modest despite high specificity. In a prospective study including more than 570 children, ultrasound demonstrated a specificity of approximately 93% but a sensitivity of only around 44% for detecting perforation [23]. Combining multiple sonographic findings into constellations can increase specificity further, reaching up to 99.5% in one series, although sensitivity for individual and combined signs has generally been reported to remain lower than specificity [24]. The pattern observed here—a sonographic sign that contributes to discrimination but does not reliably rule perforation in or out on its own—appears consistent with published data on the sonographic detection of perforation in pediatric appendicitis [23,25].

Appendix visualization was recorded with almost identical frequency in the two groups (66.7% versus 66.1%; odds ratio 1.03, 95% CI 0.39–2.85). This null result runs against the direction reported elsewhere. Perforation is listed among the recognized causes of failed sonographic identification of the appendix [36], and in a pediatric series that compared the two groups directly, the appendix was visualized in 50% of complicated versus 90% of uncomplicated cases, with non-visualization emerging as an independent predictor of complicated disease [27]. An older series nonetheless identified the inflamed appendix or an associated collection in 91.7% of patients with perforation, albeit without a non-perforated comparison group [37].

Two explanations are compatible with the discrepancy and the present data cannot separate them. The first is chance: with 27 events, the confidence interval reported here does not exclude an association of the magnitude described in [27]. The second is measurement: visualization was abstracted from narrative reports issued without a structured template, and a report that did not state whether the appendix had been seen was coded as non-visualization, which would attenuate any true difference. This null finding should not be read as evidence against the association reported elsewhere.

Appendiceal wall thickening was observed with similar frequency in perforated and non-perforated cases, indicating no evidence of discriminative ability in this cohort. A plausible explanation lies in the composition of the sample: all 142 patients had histopathologically confirmed appendicitis, and wall thickening is generally regarded as a sonographic feature of appendiceal inflammation rather than of perforation specifically. A sign present in a substantial proportion of inflamed appendices is unlikely to discriminate within a cohort in which every patient has an inflamed appendix.

An appendicolith was identified in four patients across the entire cohort (2.8%), and the resulting estimate is statistically uninformative; the extreme width of the confidence interval reflects the near-absence of the recorded sign rather than evidence of no association. This observed prevalence is considerably lower than that reported in pediatric series, but the comparison is not like for like. The figure most often quoted, 32.5%, derives from computed tomography performed in a selected subgroup of patients [38], and a further series reporting 37.6% combined radiological with intraoperative findings [39]; appendicolith presence has been associated with perforation and other features of complicated disease [40]. Ultrasound is substantially less sensitive than computed tomography for appendiceal calcifications, and an appendicolith not explicitly mentioned in a narrative report was coded as absent. The recorded frequency should therefore be interpreted as the proportion of examinations in which an appendicolith was explicitly reported on ultrasound, and no reliable conclusion regarding its relationship with perforation can be drawn from these data.

### 4.4. Comparison with the Pediatric Appendicitis Score

The Pediatric Appendicitis Score showed the highest discriminative performance among the individual parameters examined, and the difference between the combined model and the score did not reach statistical significance (Table 4). The confidence interval is compatible with a small-to-moderate advantage for either measure; the study was not designed or powered to demonstrate equivalence, and no such conclusion is drawn.

The area under the curve is, however, the wrong summary for the use proposed here. A measure intended to exclude perforation is used at a single threshold in the high-sensitivity region, and two measures with similar areas can behave very differently there. At the matched sensitivity reported in Section 3.4, the model classified substantially more non-perforated children as low risk than the score did, whereas at its own optimal threshold the score missed nine of the 27 perforations against one for the model. The two measures are therefore close in overall discrimination but not interchangeable in the operating region relevant to exclusion, and it is this difference that would have to justify the additional measurements the model requires.

The score was originally developed for the diagnosis of acute appendicitis rather than for the prediction of perforation, and its application to the present outcome lies outside its intended purpose [29]. It was derived in a prospective cohort of 1170 children aged 4–15 years to distinguish appendicitis from non-appendicitis abdominal pain [29]. Neither the derivation study nor the main validation reports have addressed perforation as a primary outcome, and no dedicated perforation-focused validation studies were identified in the sources cited here.

Two further considerations are relevant to this comparison. First, the score and the model are not informationally independent: the Pediatric Appendicitis Score incorporates leukocytosis and neutrophilia among its components [29], and the neutrophil-to-lymphocyte ratio is derived from the same cell populations. The comparison is therefore between two measures that share part of their informational basis, rather than between wholly distinct approaches.

Second, the score was reconstructed retrospectively from the medical records rather than recorded prospectively at the time of clinical assessment. Its clinical components depend on documented physical examination findings, whose completeness and consistency may vary between examiners and over time, so the reconstruction is subject to misclassification of score components [41]. The direction of the resulting bias cannot be established from these data: non-differential misclassification typically biases measures of association towards the null [41], but non-differentiality cannot be assumed here, since children with perforation were more severely ill and are likely to have been examined and documented more completely. The value obtained should therefore be read as the discrimination achieved by the score as reconstructed from these records, without any claim as to how it would compare with prospective application.

In this cohort, and with the score reconstructed as described, the two approaches were close in overall discrimination, and such advantage as the model showed lay in the operating point rather than in the area under the curve.

### 4.5. Calibration and Internal Validity

Calibration was adequate on every measure available in the development sample. These results carry less weight than their face value suggests: the Hosmer-Lemeshow test has limited power at 27 events, an absence of detected misfit is not evidence of good fit, and the calibration curve and the mean absolute error are apparent measures computed in the same data used for fitting.

The quantity that does address optimism directly is the bootstrap-corrected calibration slope, which indicates that the coefficients would require approximately 8% shrinkage if the model were applied to new patients from the same population. This slope pertains to the unpenalized fit used for resampling; the primary penalized model is already shrunken by construction, so the value is best read as a conservative bound on its calibration rather than as a shrinkage requirement. That the corresponding optimism in discrimination was small, even when the selection of the sonographic sign was repeated within each replicate, is consistent with the sample size calculation reported in Section 2.6.2 [31]. The principal departure from the ideal calibration line occurred at low predicted probabilities, in a range that spans the operating threshold, where the model underestimated the observed proportion; this reflects a local fluctuation of the smoothed curve over a sparsely populated region rather than systematic miscalibration, and the apparent and bias-corrected curves were almost identical throughout.

### 4.6. Limitations

Several limitations should be considered. This was a retrospective, single-center study covering 15 months. Perforation was taken from routine histopathology reports, without independent re-review or standardized sampling, so a focal perforation outside the examined sections would have been classified as a non-event; the 21 gangrenous specimens, a category adjacent to perforation both histologically and clinically, were likewise treated as non-events. This histopathological definition is narrower than the macroscopic criterion used in most published series, which limits direct comparison with them. With 27 events the model was restricted to three predictors, corresponding to an effective events-per-variable ratio of 4.5 once the four screened sonographic signs are counted; although the fixed ten-events-per-variable rule lacks a strong theoretical basis [42,43] and penalized estimation was used to mitigate small-sample bias [44,45], the confidence intervals around the individual odds ratios remain wide. Because the sonographic predictor was selected using the outcome and the operating threshold was optimized within the same data, the reported performance should be read as an upper bound despite bootstrap correction; thresholds chosen in this way are optimistic by construction [46,47], and the sensitivity of 96.3% rests on 26 of 27 events. Free peritoneal fluid was recorded as a single binary variable extracted from narrative reports, without distinguishing simple from complex fluid, and examinations whose reports did not mention fluid were coded as negative; ultrasound is in addition operator-dependent [48], so the predictor is a property of the report rather than of the patient. The Pediatric Appendicitis Score was reconstructed retrospectively and is subject to misclassification of its components. Symptom duration, a recognized predictor of perforation [49], and time from presentation to surgery were not available and could not be adjusted for. All patients had confirmed appendicitis and had already been selected for laparoscopic appendectomy, so the predictive values apply only within that spectrum; 36 children who underwent primary open appendectomy were not analyzed, and if that approach was chosen more often in advanced presentations, the analyzed sample is depleted of severe cases. Finally, the model has not been evaluated in an independent cohort and is not presented as a validated prediction tool.

Overlap with a previous report. The same cohort has been analyzed in a companion report published in Surgeries [50], with different outcomes and a different modelling strategy, as detailed in Section 2.1. The descriptive characterization of the cohort is therefore shared between the two reports, and because the outcomes are related but not identical, estimates from the two should not be pooled when synthesizing evidence.

## 5. Conclusions

In children operated for acute appendicitis at a single center, two routinely available inflammatory markers and one sonographic finding were each independently associated with histopathologically confirmed perforation, and their combination discriminated well, with limited optimism on internal validation and adequate calibration. The practical implication is asymmetric: at a threshold set for high sensitivity, 1 of the 87 children classified as low risk had perforation, whereas fewer than half of those above the threshold did. The combination is therefore informative for excluding perforation rather than for establishing it, and its intended moment of use is after the decision to operate has been taken, where anticipating a perforated appendix can inform the choice and duration of empirical antibiotic therapy, the urgency and staffing of the operation, and the information given to families, rather than whether an operation is indicated. Because the sonographic predictor was selected within these same data and the threshold was optimized in-sample, the reported performance should be read as an upper bound, and external validation using structured sonographic reporting is required before clinical use can be considered.

## Figures and Tables

**Table 1:** Baseline demographic, laboratory, and sonographic characteristics of the study cohort, stratified by histopathologically confirmed appendiceal perforation.

Variable	Non-Perforated (n = 115)	Perforated (n = 27)	*p*	SMD
Demographic and anthropometric variables				
Age (years)	11.00 [9.00–14.00]	10.00 [7.50–14.00]	0.605	0.104
Female sex, n (%)	45 (39.1)	10 (37.0)	1.000	0.043
Body mass index (kg/m^2^)	19.39 [16.53–22.21]	21.13 [15.99–22.84]	0.662	0.150
Laboratory parameters				
Leukocytes (×10^9^/L)	13.40 [9.04–16.74]	14.88 [13.77–17.64]	0.023	0.532
Neutrophils (×10^9^/L)	10.80 [5.80–13.27]	12.57 [11.49–15.44]	0.004	0.652
Neutrophil-to-lymphocyte ratio	5.25 [2.80–9.53]	9.91 [7.33–13.50]	<0.001	0.820
C-reactive protein (mg/L)	9.40 [1.70–34.50]	89.20 [46.85–130.00]	<0.001	1.241
Clinical score				
Pediatric Appendicitis Score	7.00 [6.00–8.00]	9.00 [8.00–10.00]	<0.001	1.576
Sonographic findings				
Appendix visualized, n (%)	76 (66.1)	18 (66.7)	1.000	0.012
Appendiceal wall thickening, n (%)	54 (47.0)	12 (44.4)	0.834	0.050
Free peritoneal fluid, n (%)	19 (16.5)	13 (48.1)	0.001	0.718
Appendicolith, n (%)	3 (2.6)	1 (3.7)	0.574	0.063

Note: Continuous variables are presented as median [interquartile range] and were compared using the Mann–Whitney U test; given that all continuous variables except the leukocyte count deviated significantly from normality on Shapiro–Wilk testing, all were summarized uniformly for consistency of presentation. Categorical variables are presented as n (%). Sex was compared using the chi-squared test with continuity correction. The four sonographic signs were compared using Fisher’s exact test, applied uniformly across this group of variables because one sign (appendicolith) had expected cell counts below five; the odds ratios reported in Section 3.2 derive from the conditional maximum likelihood estimator implemented in that test. The Pediatric Appendicitis Score was available for 140 of 142 patients (98.6%) and was summarized on the available cases. SMD, standardized mean difference.

**Table 2:** Multivariable Firth penalized logistic regression model for the prediction of appendiceal perforation.

Predictor	β	SE	OR (95% CI)	OR per Doubling (95% CI)	*p*
Intercept	−7.190	1.297	0.001 (0.000–0.008)	—	<0.001
log-NLR	1.114	0.417	3.045 (1.381–7.547)	2.164 (1.251–4.059)	0.005
log-CRP	0.949	0.218	2.583 (1.733–4.213)	1.931 (1.464–2.710)	<0.001
Free peritoneal fluid	1.291	0.556	3.638 (1.215–11.375)	—	0.021

Note: Firth’s penalized maximum likelihood logistic regression; n = 142, 27 events, nominal events per variable = 9.0 and effective events per variable = 4.5 after accounting for the four sonographic signs screened (Section 2.6.2). Confidence intervals and *p*-values were derived from profile penalized likelihood. The two inflammatory markers were specified a priori; the sonographic predictor was selected from the four recorded signs on the basis of the exploratory analysis reported in Section 3.2. No automated or stepwise selection was applied. Odds ratios for log-transformed predictors were calculated as exp(β × ln 2) and are reported per doubling of the original variable to facilitate clinical interpretation. Likelihood ratio test χ^2^ = 50.45, df = 3, *p* < 0.001. The Brier score (0.093) was computed directly from the predicted probabilities of the penalized model. Nagelkerke R^2^ (0.502) was obtained from the corresponding unpenalized maximum likelihood model fitted to the same predictors, since this measure is not returned by the penalized-likelihood implementation used here; given the close agreement between the penalized and unpenalized fits (Table S1), it is taken to describe the penalized model to a close approximation. β, regression coefficient; CI, confidence interval; CRP, C-reactive protein; NLR, neutrophil-to-lymphocyte ratio; OR, odds ratio; SE, standard error.

**Table 3:** Diagnostic performance of individual preoperative parameters and of the combined model for the prediction of appendiceal perforation.

Parameter	AUC (95% CI)	Cut-Off	Se (%)	Sp (%)	PPV (%)	NPV (%)
Combined model	0.900 (0.839–0.963)	0.120	96.3	74.8	47.3	98.9
Pediatric Appendicitis Score (n = 140)	0.855 (0.774–0.936)	≥8.5	66.7	92.0	66.7	92.0
C-reactive protein	0.842 (0.763–0.920)	≥65.2 mg/L	70.4	87.8	57.6	92.7
Neutrophil-to-lymphocyte ratio	0.742 (0.652–0.832)	≥9.66	70.4	75.7	40.4	91.6
Free peritoneal fluid	0.658 (0.556–0.760)	Present	48.1	83.5	40.6	87.3

Note: Optimal cut-off values were determined by maximizing Youden’s index (sensitivity + specificity − 1). Cut-offs for C-reactive protein and the neutrophil-to-lymphocyte ratio are presented on the original scale; the corresponding values on the logarithmic scale used in the model were 4.193 and 2.268, respectively. The cut-off for the combined model refers to the predicted probability of perforation, computed from the penalized model. The Pediatric Appendicitis Score was analyzed on available cases (n = 140) and was not included in the multivariable model. PPV and NPV were calculated at the observed perforation prevalence of 19.0% and will differ in populations with a different prevalence. At a common operating point defined by the combined model’s Youden-optimal sensitivity of 96.3%, the corresponding specificities were 74.8% for the combined model, 50.4% for C-reactive protein, 44.3% for the neutrophil-to-lymphocyte ratio and 38.9% for the Pediatric Appendicitis Score; these matched-sensitivity operating points were computed post hoc within the development sample and are reported as exploratory. AUC, area under the receiver operating characteristic curve; CI, confidence interval; NPV, negative predictive value; PPV, positive predictive value; Se, sensitivity; Sp, specificity.

**Table 4:** Pairwise comparison of areas under the curve between the combined model and individual parameters (DeLong’s test).

Comparator	ΔAUC (95% CI)	*Z*	*p*
Pediatric Appendicitis Score	0.044 (−0.053 to 0.141)	0.888	0.375
C-reactive protein	0.059 (0.009–0.109)	2.295	0.022
Neutrophil-to-lymphocyte ratio	0.158 (0.067–0.250)	3.396	<0.001
Free peritoneal fluid	0.242 (0.150–0.335)	5.125	<0.001

Note: Pairwise comparisons of correlated ROC curves were performed using DeLong’s test. The comparison with the Pediatric Appendicitis Score was restricted to patients with an available score (n = 140), for which the combined model yielded an AUC of 0.899. All 27 outcome events were included in this comparison; the two patients with a missing score were both non-perforated. A positive ΔAUC indicates higher discrimination for the combined model. The three comparisons against the model’s own components are shown for completeness; because the model was fitted to those predictors in the same sample, it discriminates at least as well as each by construction, and their *p*-values do not test an independent hypothesis. The absence of a statistically significant difference versus the Pediatric Appendicitis Score should not be interpreted as evidence of equivalence, as the study was not powered for equivalence testing and the confidence interval was wide. ΔAUC, difference in area under the curve; CI, confidence interval.

## Data Availability

The data presented in this study are available on request from the corresponding author. The data are not publicly available due to restrictions eg privacy or ethical.

## Supplementary Materials

The following supporting information can be downloaded at: https://www.mdpi.com/article/10.3390/children13091152/s1. Table S1: Conventional (unpenalized) maximum likelihood logistic regression model for the prediction of appendiceal perforation, fitted to the same data as a sensitivity analysis; File S1. STROBE Statement—Checklist of items that should be included in reports of cohort studies.

## Abbreviations

The following abbreviations are used in this manuscript:

ASA	American Society of Anesthesiologists
AUC	Area under the receiver operating characteristic curve
CI	Confidence interval
CRP	C-reactive protein
EPV	Events per variable
NLR	Neutrophil-to-lymphocyte ratio
NPV	Negative predictive value
OR	Odds ratio
PAS	Pediatric Appendicitis Score
PPV	Positive predictive value
SMD	Standardized mean difference
STROBE	Strengthening the Reporting of Observational Studies in Epidemiology
