# Supplementary material for: Preoperative Inflammatory Markers and Sonographic Free Peritoneal Fluid in Relation to Histopathologically Confirmed Appendiceal Perforation in Children: A Retrospective Cohort Study
Source: Children (Basel). 2026 Aug 27;13(9):1152. doi: 10.3390/children13091152 (PMC13604985; doi:10.3390/children13091152)
Supplement: Supplementary file 1 [file children-13-01152-s001.zip › Table S1.pdf]

Supplementary Materials

Preoperative Inflammatory Markers and Sonographic Free Peritoneal Fluid in Relation to Histopathologically Confirmed Appendiceal Perforation in Children: A Retrospective Cohort Study

Table S1. Conventional (unpenalized) maximum likelihood logistic regression model for the prediction of appendiceal perforation, fitted to the same data as a sensitivity analysis.

**Predictor**	β	**SE**	**OR (95% CI)**	p
Intercept	−7.709	1.419	0.000 (0.000–0.007)	<0.001
log-NLR	1.196	0.447	3.307 (1.378–7.937)	0.007
log-CRP	1.021	0.236	2.776 (1.748–4.409)	<0.001
Free peritoneal fluid	1.354	0.586	3.872 (1.229–12.204)	0.021

Note: Conventional maximum likelihood logistic regression fitted to the same cohort and the same three predictors as the primary penalized model reported in Table 2 of the main text; n = 142, 27 events, nominal events per variable = 9.0 (effective 4.5; see main text Section 2.6.2). Confidence intervals and p-values were derived from Wald statistics, in contrast to the profile penalized likelihood used for the primary model. This model is presented as a sensitivity analysis to assess the stability of estimation, and was also the model to which the bootstrap internal validation was applied, as described in Section 2.6.4 of the main text. Relative to this unpenalized fit, the penalized estimates were attenuated towards the null by 4.6% to 7.1% on the coefficient scale (6.9% for log-NLR, 7.1% for log-CRP, and 4.6% for free peritoneal fluid), with no change in the direction or statistical significance of any association, which is the expected consequence of penalization and indicates that estimation was stable in this dataset. Model fit (unpenalized): Nagelkerke R^2 = 0.502; apparent AUC = 0.900. β, regression coefficient; CI, confidence interval; CRP, C-reactive protein; NLR, neutrophil-to-lymphocyte ratio; OR, odds ratio; SE, standard error.
